# Analysis of Cluster and Unrest Behaviors of Laying Hens Housed under Different Thermal Conditions and Light Wave Length

**DOI:** 10.3390/ani11072017

**Published:** 2021-07-06

**Authors:** Aline Mirella Fernandes, Diogo de Lucca Sartori, Flávio José de Oliveira Morais, Douglas D’Alessandro Salgado, Danilo Florentino Pereira

**Affiliations:** 1 Graduate Program in Agribusiness and Development, School of Science and Engineering, São Paulo State University, Tupã 17602-496, Brazil; alinemirellafernandes@gmail.com; 2 Department of Biosystems Engineering, School of Science and Engineering, São Paulo State University, Tupã 17602-496, Brazil; diogo.sartori@unesp.br (D.d.L.S.); flavio.morais@unesp.br (F.J.d.O.M.); douglas.salgado@unesp.br (D.D.S.); 3 Department of Management, Development and Technology, School of Science and Engineering, Sao Paulo State University, Tupã 17602-496, Brazil

**Keywords:** image analysis, precision poultry farming, animal welfare, movement analysis, LED, comfort index

## Abstract

**Simple Summary:**

The effects of lighting wavelength on the behavior of laying hens are not yet completely known. This study observed three groups of birds housed under different lighting colors (blue, green, and red) for 90 days. Important differences were found regarding the unrest and cluster behaviors of the birds. It was found that, at shorter wavelengths (blue light), birds became more agitated, while, at longer wavelengths (red light), birds became more clustered. When subjected to cold or heat stress, birds expressed unrest and cluster behaviors in different ways, indicating that further studies should be conducted to better clarify the effects of lighting on the behavior and well-being of laying hens.

**Abstract:**

Laying hens are affected by the intensity, wavelength, and duration of light, and the behavioral patterns of these animals are important indicators of stress. The objective of the present study was to evaluate cluster and unrest behaviors of lying hens submitted to three environments with different treatments of monochromatic lighting (blue, green, and red). For 29 weeks, 60 laying hens from the Lohmann variety were divided into three groups and monitored by surveillance cameras installed on each shed ceiling and directed to the floor. Each group was housed in a small-scale shed and maintained under a monochromatic lighting treatment. The recordings were made at two times of the day, 15 min in the morning and 15 min in the afternoon, and the videos were processed, segmented, and analyzed computationally. From the analysis of the images, the cluster and unrest indexes were calculated. The results showed the influence of lighting on these behaviors, displaying that the birds were more agitated in the treatments with shorter wavelengths. Cluster behavior was higher in birds housed under red light. There was an interaction between the lighting treatments and the thermal environment, indicating that more studies should be carried out in this area to better understand these behavioral changes.

## 1. Introduction

The use of artificial lighting in the breeding of laying hens is essential to achieve the necessary illuminance, spectrum of light, and suitable photoperiod for the physiological stimulation of the animals [1]. Thus, lighting has a great influence on the productivity of these animals and is a factor of high importance for the welfare of birds confined in conventional egg production systems.

Well-being is defined as the animal’s ability to interact and live well in its environment [2]. Light is an essential factor in the microclimate of the poultry house, which means variations in its distribution, intensity, wavelength, and duration affect the welfare and performance of birds [3,4,5,6,7].

The lighting period can contribute to normal and healthy behavior patterns [4,8]. Broilers are more active when in contact with high illuminance (180–200 lux) [9]. For broiler chickens, longer wavelengths (orange and red) make these birds more agitated and aggressive [4].

Behavior is an indicator of stress, and it is affected by the wavelength of light [10,11,12,13,14,15,16,17]. Broilers move more under long-wavelength lighting and tend to stay seated and stationary for longer in environments with blue and green light [5]. Aggressive behavior can be controlled by decreasing the light intensity or using different wavelengths [18]. However, knowledge about the effects of different wavelengths on laying hens is still limited [7].

Birds have four types of single cones, double cones, and rods [19]. Olsson and colleagues [20] report that single-cone photoreceptors are responsible for color vision, each sensitive to a range of specific wavelengths. The maximum sensitivity of these cones is for long wavelengths (L, red) 571 nm, medium wavelengths (M, green) 508 nm, short wavelengths (S, blue) 455 nm, and very short wavelengths (VS, ultraviolet) 415 nm [21]. When a thermal environment changes from thermoneutrality to heat or cold stress, the behavior of birds, whether individual or collective, occurs more quickly in order to mitigate its effects [22,23,24].

When birds are subjected to heat stress situations, several changes in energy metabolism start to occur, altering thermoregulatory and behavioral responses, and part of the energy that would be used for egg production is redirected to maintain the bird’s homeostasis [12,25]. In this situation, one can observe increased water intake, reduced feed consumption, increased respiratory rate, and behaviors such as aggressive pecking and wing exposure as a way to dissipate endogenous heat and maintain homeostasis [26,27,28,29].

The behavioral observation of animals can be performed by a human being present at the place where the animals are housed. However, this is a time-consuming, expensive, subjective, and error-prone method. Automated monitoring, through digital cameras, has the ability to generate data that provide an objective measure of behavior, without disturbing animals [30]. In addition to being a low-cost technology, it enables the monitoring of animal behavior on an automated [31,32,33], non-invasive [34], and ongoing basis.

Digital cameras have been used to monitor the behavior of birds, in which the images analyzed use computer vision techniques [32,35,36,37]. Computer vision is responsible for extracting relevant information based on images captured by digital cameras, whether through photographic or video images, sensors, and other devices [38]. These technologies have shown great evolution over the past few years [39].

The cluster behavior of laying hens can be classified automatically through image analysis [34]. Pereira and co-workers [34] found that, in conditions of lower temperatures, the laying hens agglomerate more, suggesting that this group behavior can be used to estimate bird thermal comfort. The evaluation of laying hens’ agitation behavior was proposed by [40], through an unrest index calculated from image analysis. This index was used to estimate bird thermal comfort, and the authors found that, in high temperature conditions, birds moved less in the poultry house. The combined use of these methods can contribute to a more accurate assessment of the conditions and well-being of commercial birds at their breeding place.

The objective of this work was to evaluate the cluster and unrest behaviors of laying hens in different thermal conditions (cold, comfort, and heat), submitted to three different monochromatic lighting sources (blue, green, and red) in order to verify whether the wavelength of the light source influences these behaviors.

## 2. Materials and Methods

The study was carried out at the facilities of the Bioterium of the School of Sciences and Engineering, from the São Paulo State University (UNESP), in the city of Tupã, Brazil. The experiments were carried out for 90 days, from 10 June to 8 September 2020, in which the first seven days were dedicated to the adaptation of the birds to the new environment and accommodation conditions.

### 2.1. Description of Birds and Facilities

For this study, 60 laying hens of the Lohmann variety were monitored at, initially, 29 weeks of age. At the beginning of the experiments, the birds, which were obtained from a commercial farm, were randomly divided into three groups of 20 birds each. Food was administered daily, in the amount of 110 g/bird, once a day, in the morning. Access to water was ad libitum, through nipple drinkers. The light management was similar to that adopted by the original farm, with a photoperiod of 17 h of light.

**Figure 1:**
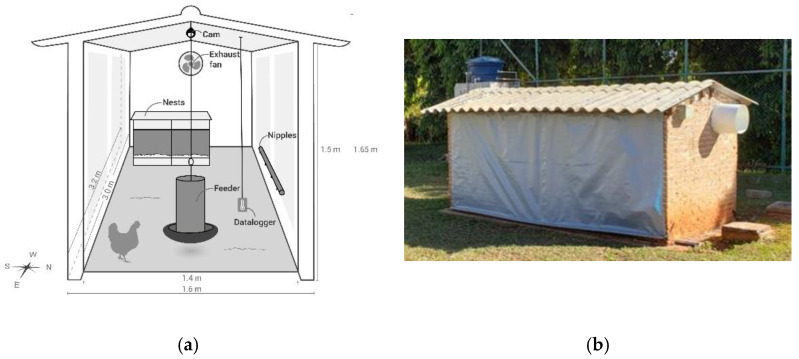
Experimental houses used in the study: (**a**) layout of the nests, feeders and drinking fountains inside the sheds; (**b**) external view of an sheds.

Three models of sheds were used on a reduced scale, arranged in an east–west orientation, where the birds were housed in a 15 cm high shavings bed. Each poultry house had two 40 × 40 × 40 cm^3^ box-type nests installed, a pendular feeder (Φ 30 cm), and four nipple drinkers, as shown in Figure 1a.

The sheds were completely sealed with the use of plastic sheeting, to prevent external lighting from influencing the internal lighting system of each environment. For ventilation control and air renewal inside the sheds, exhaust fans (Φ 30 cm, 120 W, and outflow 30 m^3^/min) were installed on one of the longitudinal walls (Figure 1b).

**Figure 2:**
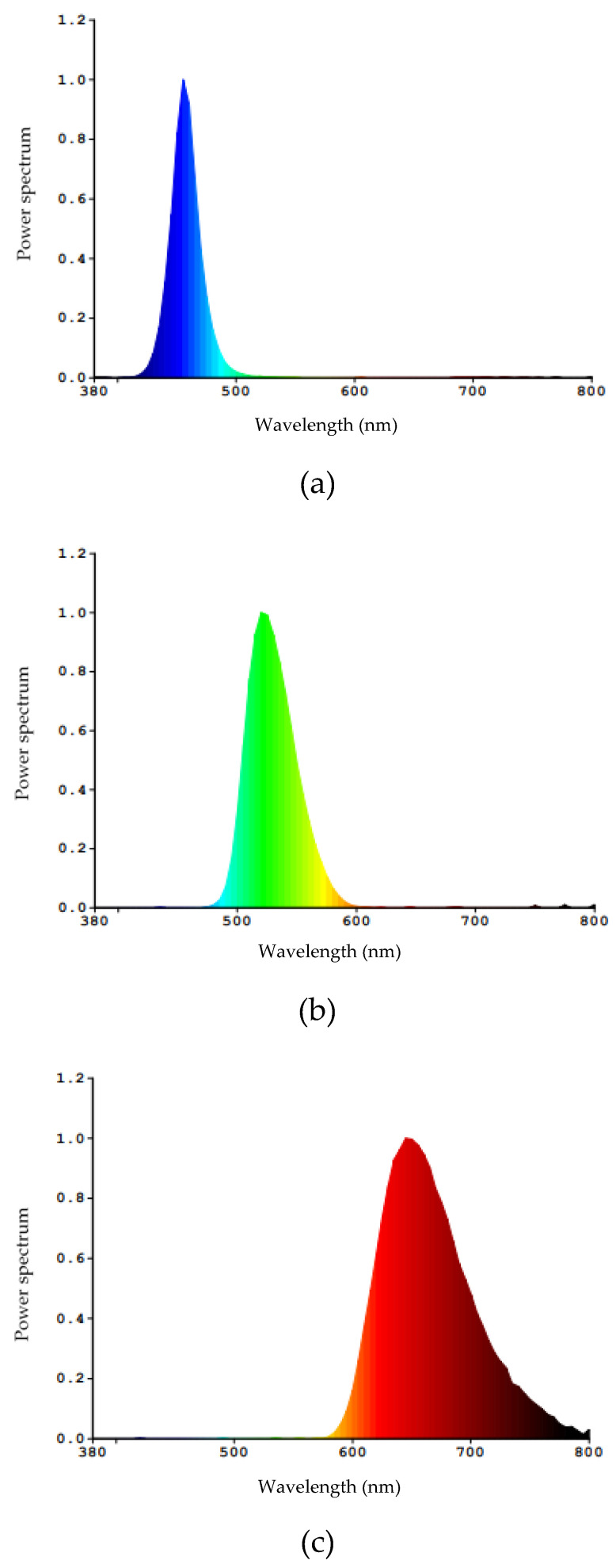
Luminous spectra of the lamps used in the experiment: (**a**) Model TKL Colors—Blue; (**b**) Model TKL Colors—Green; (**c**) Model TKL Colors—Red. (Data provided by the manufacturer).

In each of the sheds, the group of birds was exposed to the treatment of monochromatic blue, green, and red LED lighting. (Initially, the experimental design provided for a control treatment, where 20 birds were housed under white light. However, in this treatment there was an outbreak of *Lipeurus caponis* lice that affected the behavior of the birds, and the data could not be used in this work). Each light source had a different light spectrum, as shown in Figure 2. The number of lamps in each treatment was calculated from the characteristics of the lamps provided by the manufacturer, to provide the same 100 lux illuminance, similar to the methodology used by Zupan [41].

### 2.2. Monitoring of the Thermal Environment

In order to monitor the internal thermal environment of the sheds, a datalogger of the HOBO^®^ brand, model U12-012, was installed. The datalogger was positioned in the center of the sheds, at the same height as that of the birds, to record data on temperature and relative humidity. This equipment was programmed to record the temperature and relative humidity of the air at 5 min intervals for 24 h throughout the entire observational study.

From the temperature and relative humidity data, the Temperature and Humidity Index (*THI*) was calculated for each shed internal environment, using Equation (1), described by [42] and used for birds by [43].

(1) $$THI=0.8\times T+\frac{RH\times \left(T-14.3\right)}{100}+46.3$$

where: *T* = temperature dry bulb in °C; *RH* = relative humidity of the air (%).

### 2.3. Bird Monitoring System

The birds were monitored by digital surveillance cameras, which were installed in the center of the shed ceiling and directed to the floor, at a 1.5 m height. The cameras recorded for 15 min in the morning and 15 min in the afternoon, according to the methodology used by [44,45]. The captured images were recorded and stored in video format using Digital Video Recorder (DVR) equipment.

The transmission of the images from the cameras to the DVR was made by coaxial cables. The video cameras installed were from the POWER^®^ brand, model AP2688W, with a Charge-Coupled Device (CCD) analog image sensor. The resolution was that of 352 × 240 pixels, a lens with a focal length of 2.8 mm, a viewing angle of 60°, and video standard NTSC (National Television System(s) Committee). The DVR equipment was the model VD 4E120 of the Intelbras^®^ brand, with the Linux operating system installed, supported video format NTSC, had a video recording speed of 30 frames per second (fps) and with capacity for 4 video channels and support for 1 1TB SATA HD.

**Figure 3:**
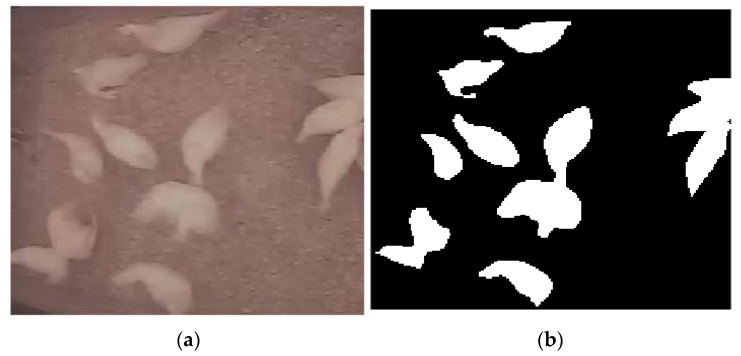
Example of segmentation of an image: (**a**) Original cropped image; (**b**) segmented image.

From the framing obtained by the cameras inside each shed, an area free of objects and equipment was defined, so that the activity of the birds could be monitored. This area was delimited in the first frame and replicated for all consecutive frames of all the video files that composed the samples. The images were processed and analyzed using MATLAB^®^ software. In the image processing, low-pass filters and threshold-based segmentation techniques were applied so that only the birds were highlighted (Figure 3).

From the segmented (binarized) images, it was possible to extract measures used to calculate the cluster and unrest indexes, used in this work to describe the group behavior of the birds in each treatment.

### 2.4. Measures of Cluster and Unrest Indexes

Two indicators were used to describe the group behavior of birds: the cluster index, described by [37] and the unrest index, described by [40]. The cluster behavior is characterized by the reduction in distances between individuals and the pillaging of these birds. On the other hand, the unrest behavior is associated with the movement of the flock in the experimental house.

With the segmented image, the positions of the centers of mass of the birds (or groups of birds) were recorded in each video frame, in addition to the area and the perimeter of the shapes that the birds assume. These measures were used to calculate the cluster index described by Equation (2).

(2) $$Cluster\;Index_{i}=\frac{2\times \bar{A}\times \sqrt{h^{2}+w^{2}}}{\bar{P}\times \bar{D}\times n_{A}}-1$$

where:$\;Cluster\;Index_{\left(i\right)}$ is the cluster index of the birds observed in the ith frame of the video; $\bar{A}$ and $\bar{P}$ are, respectively, the average area and perimeter (in pixels) of the shapes observed in the frame; $\bar{D}$ is the average distance between the centers of mass of the shapes in the scene;$\;n_{A}$ is the number of clusters, and h and w correspond to height and width (in pixels) of the cropped image.

For the calculation of the unrest index (measured in centimeters), initially, the distances from the birds’ centers of mass in one frame, at time *i−*1, were calculated to the birds’ centers of mass in the next frame, at time *i*. From the distance measurements between the centers of mass of the birds between the frames, the Hausdorff distance was extracted, which is the mathematical measure that represents the distance between two sets. The Hausdorff distance makes up the unrest index, as described by Equation (3).

(3) $$Unrest\;Index_{\left(i,i-1\right)}=k.max\left\{dH\left(F_{\left(i\right)},F_{\left(i-1\right)}\right),\;dH\left(F_{\left(i-1\right)},F_{\left(i\right)}\right)\right\}$$

where:$\;Unrest\;Index_{\left(i,\;i-1\right)}$ is the unrest index (cm) of the birds between two frames recorded with 1 (one) second difference; *i* is the position of the frame in the video; $F_{\left(i\right)}$ is the current frame; $F_{\left(i-1\right)}$ is the previous frame; dH is the Hausdorff distance between group of birds from one frame to the other, and k is the proportionality factor calculated by Equation (4).

(4) $$k=\frac{2H\tan\left(\alpha /2\right)}{w}$$

where: k is the proportionality factor; *H* is the height (cm) of the installed camera in relation to the floor; α is the opening angle of the camera lens, and w is the length (pixels) of the CCD sensor, which corresponds to the length of the largest measurement of the frame captured by the camera.

### 2.5. Analysis

This is considered an observational cohort study, as it followed three groups of similar individuals (cohorts) under different environmental treatments. Treatments were under blue, green, or red lighting conditions in each experimental house.

In this study, agitation and agglomeration behaviors were compared using the unrest index and cluster index, respectively. Initially, exploratory analyses were performed through graphical interpretations, and later confirmatory analyses through the analysis of variance and the multiple means comparisons test.

## 3. Results and Discussion

### 3.1. Thermal Environment

For laying hens, it is considered that temperature and THI values above 28 °C and 78, respectively, are considered situations in which the birds are outside the thermal comfort zone and, therefore, already characterize heat stress [43]. On the other hand, temperatures below 15 °C and a THI below 59 are considered to induce cold stress [46].

**Figure 4:**
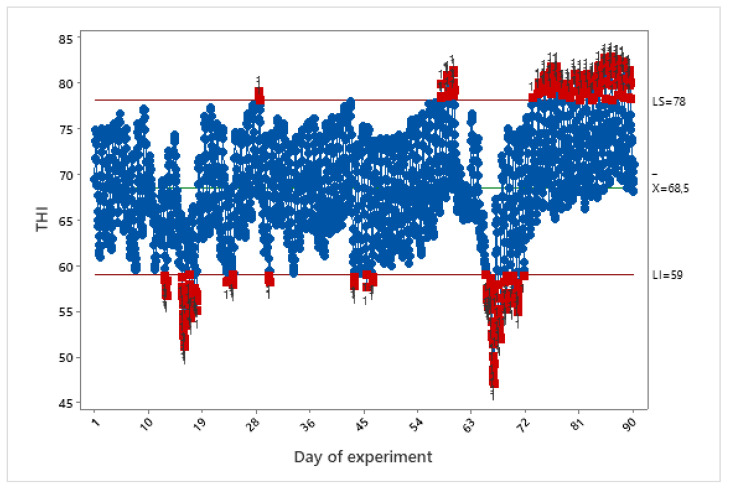
THI averages calculated for each hour, during the period of the observational study.

Figure 4 shows the variation of the THI for each hour during the entire period of the experiment. When the THI values of the environment are below or above the thermoneutrality limits defined in the literature, the values are highlighted in red.

Figure 4 shows that the birds were exposed to conditions of a thermoneutral environment most of the time. However, they were also exposed to conditions of cold and heat stress at some moments. Considering the recording times of the videos for behavior analysis, 49 thermoneutrality recordings, 20 heat recordings, and 15 cold recordings were obtained.

### 3.2. Behavior Analysis

Approximately 36 h of images were recorded in a video file in the three lighting treatments, which provided an analysis of about 520,000 frames, allowing the assessment of birds’ unrest and cluster behaviors through their respective indexes.

**Figure 5:**
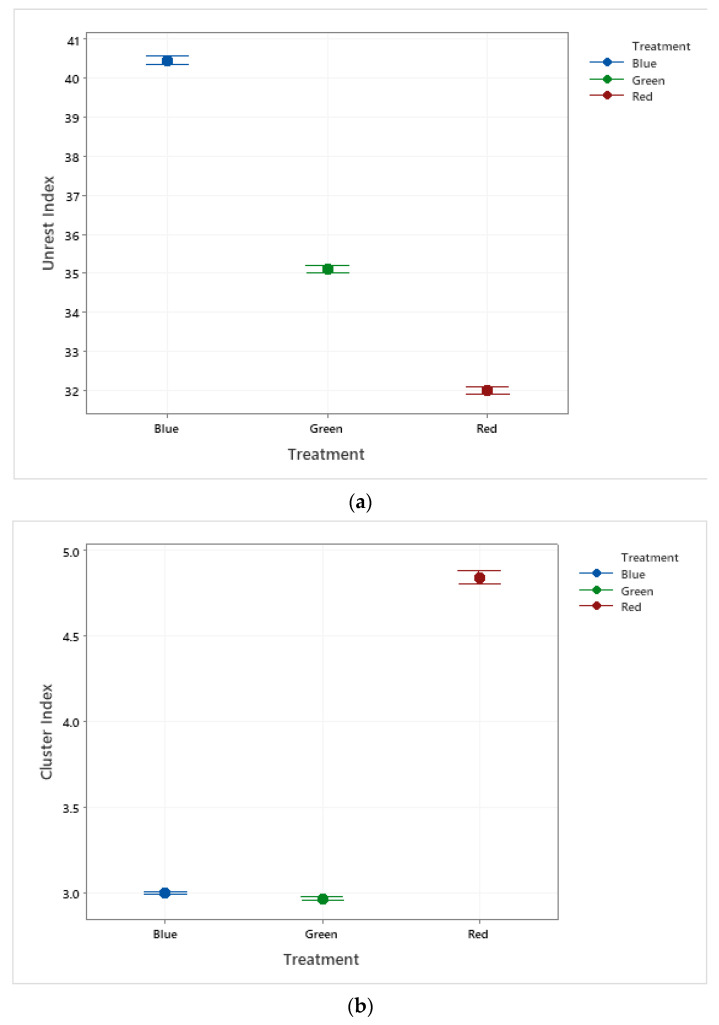
Confidence intervals of the mean for the (**a**) unrest index and (**b**) cluster index verified for the blue, green, and red light treatments.

It was observed that the birds’ unrest decreases with increasing wavelength (Figure 5a). The group of birds housed under blue lighting treatment were those that showed greater unrest behavior, compared to the other treatments. The confidence intervals for cluster behaviors between the lighting treatments, where the influence of the red wavelength is verified in the increase in the intensity of this collective behavior, are shown in Figure 5b.

For broilers, Sultana and co-workers [5] and Hesham and colleagues [47] found that the birds clustered less under blue lighting (short wavelength) and showed greater unrest when exposed to red lighting (long wavelength). In this study, laying hens exposed to red light were more crowded and less agitated when compared to green and blue lights. The results suggest that the effects of lighting wavelength promote different effects in broilers and laying hens, as also noted by Wichman and colleagues [48], or that age or sexual maturity are determinants for the choice of which light spectrum is the most suitable for each stage of production, as verified by Wei and co-workers [7] in breeding commercial poultry. Red monochromatic LED lighting reduced aggression [49] and reduced bird mortality [50], indicating that this wavelength may be associated with reduced stress. In broilers, studies have shown that, under red light, the birds are more agitated and aggressive [4], while laying hens have an increase in egg production [51] and reduction in stress [52].

**Figure 6:**
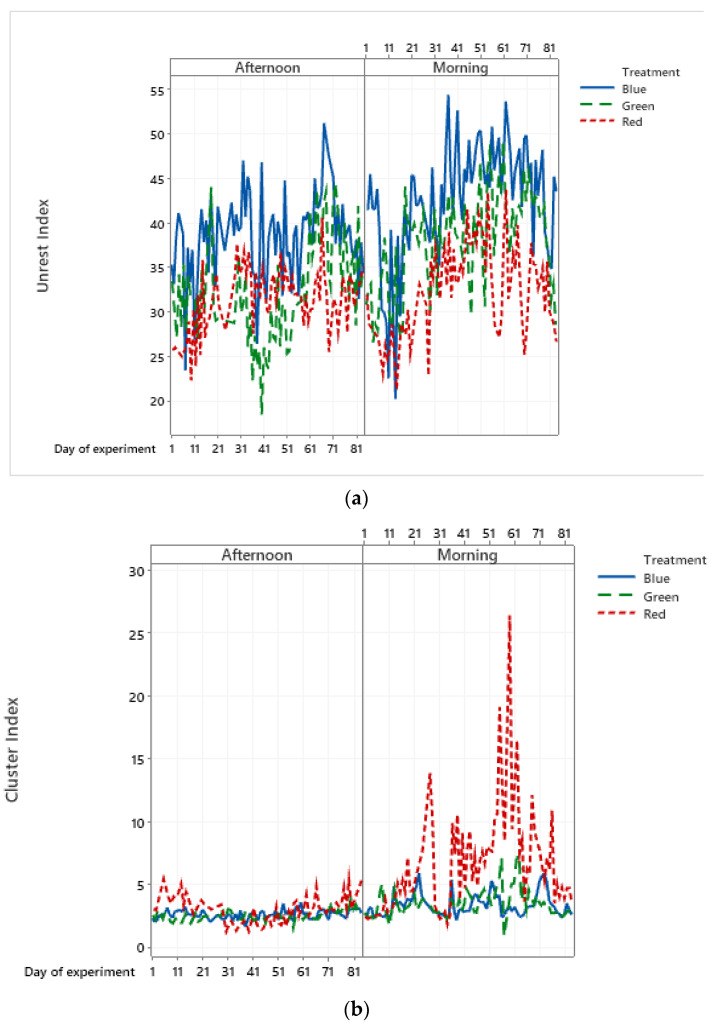
Average daily values for (**a**) unrest index and (**b**) cluster index, referring to samples of 15 min videos recorded and analyzed by period of the day.

Marino [53] describes that personality is defined by three traits (boldness, activity/exploration, and vigilance) and that bird emotions are a combination of cognitive ability and sociability. Birds are highly dependent on vision to express behaviors, especially social behaviors [54,55]. Thus, in environments with monochromatic lighting, it is expected that visual acuity is affected and that social behaviors are altered, influencing the exploration behavior and the welfare of the birds. During the experiment, it was noticeable that the birds under blue lighting were more agitated, as shown by the unrest index (Figure 6a), followed by the green and red treatments. Despite some interaction between the days, there is a tendency to reduce the unrest with the increase in the wavelength.

In the birds’ cluster behavior, a greater interaction of the cluster index was observed between the data from the blue and green light groups. However, the cluster in the group housed under red light was much greater, showing very pronounced peaks in the morning period (Figure 6b). Early in the day, there was a greater supply of food in the feeders and, revisiting the videos, it was found that this cluster occurred around the feeder, demonstrating that birds at this wavelength are more willing to eat.

Lighting is known to affect the behavior of birds [13,15,16,17]. The birds eat more when exposed to green light when compared to blue light [16]. The birds spend more time around the drinker when under blue and white light, and less time under red and green light [11]. In this work, the dwelling times in the feeder and drinker were not monitored, but the results suggest that there was a greater cluster of birds observed in the red wavelength around the feeder. Birds prefer environments with short-wave lighting (blue and green) to environments with red lighting [14]. Chickens perceive light at a different intensity than humans [56]. Photoreceptors have colored oil droplets that act as a filter for different wavelengths of light [20]. For this reason, each photoreceptor is sensitive to different wavelengths range, with violet light photoreceptors being the most sensitive, followed by blue, green, and red, in that order [19]. In this experiment, this characteristic of the birds’ vision may have affected the laying hens’ behaviors in response to the light intensity, so that, under the blue light treatment, the birds may have been hyper-stimulated, which would explain the more agitated behavior.

**Figure 7:**
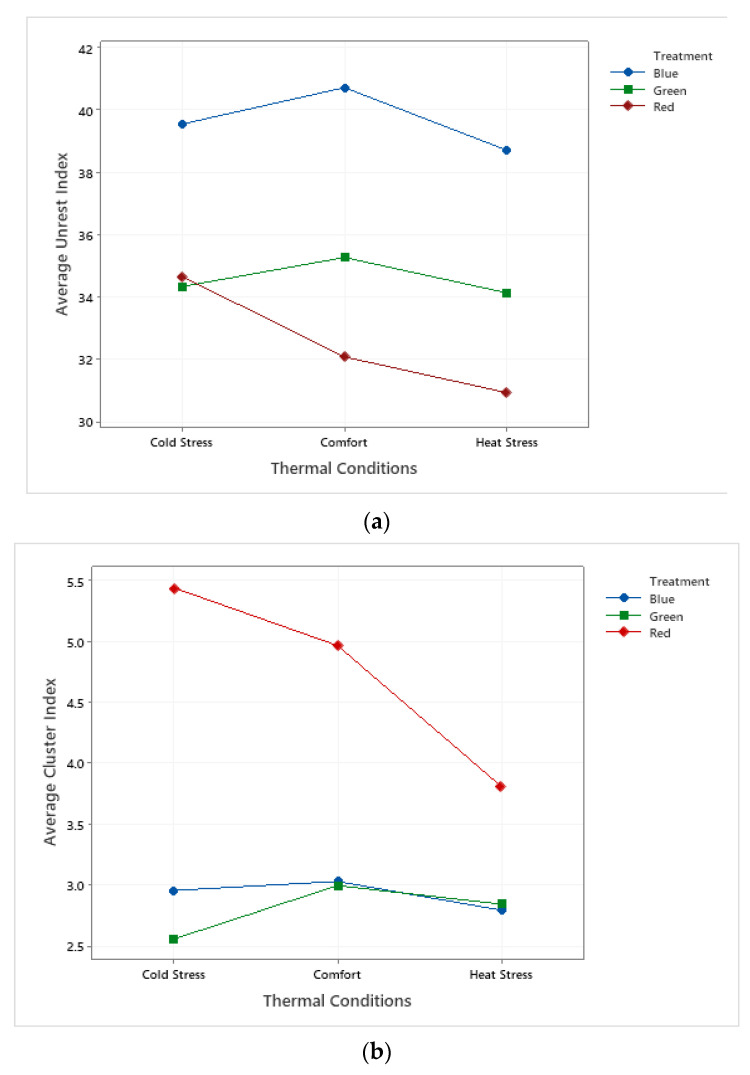
Interaction for (**a**) unrest and (**b**) cluster behaviors in relation to comfort, according to treatment type.

The interaction between the unrest and cluster behaviors of the birds was verified for the thermal conditions observed in the study (Figure 7).

While it is observed that birds reared under red light show a reduction in the unrest index as the temperature increases, birds reared under green and blue light showed a higher unrest index when the temperature was of thermal comfort. Although the results indicate this interaction, it can be seen in Figure 7a that that there is an influence of blue light in the greatest unrest in birds, for all environmental conditions, followed by green and red light, in that order. This figure also shows that, in the blue and green treatments, the unrest is greater in thermoneutrality. Under red light, there was a decrease in unrest with the increase in the wavelength, showing that light wavelength can affect the behavioral response of birds to thermal stress.

Figure 7b reinforces the evidence that the red light influences a greater willingness of birds to feed in all the thermal conditions observed in this study. There is also a tendency to reduce cluster under heat stress conditions in all treatments.

In Table 1 and Table 2, the means comparison test was applied for the crossover results of lighting treatment for periods of the day and thermal environments in which the unrest and cluster behaviors were evaluated. The results confirm the evidence presented in the previous figures.

Under high temperature conditions, birds clustered less [37] and moved less [12], corroborating the results of this study. Birds prefer to feed in the morning [57,58]. In the afternoon, they remain seated, stationary, for longer periods of time. The results of Table 2 and 3 corroborate these observations, because in the morning, there was a greater cluster of birds around the feeder, for all treatments, while in the afternoon, there was less unrest, except under the red light, where the movement of the birds was higher in the afternoon. The presence of food attracts birds to the feeder and, therefore, increases the cluster of birds around it [57,59,60]. This increase in cluster behavior at the arrival of fresh food is also associated with the common bird behavior of feeding in groups [61].

## 4. Conclusions

The different monochromatic lighting regimes affected bird behaviors of unrest and cluster. It was found that the unrest was greater under blue light, followed by green and red, which indicates that the increase in the wavelength of the light source may be associated with a lower level of expression of the unrest behavior, or even that longer wavelengths have a calming effect on laying hens. However, studies with more birds are needed to prove this hypothesis.

The interaction was verified between the lighting treatments and the thermal environment, suggesting that further studies should be carried out in this area to better under-stand these behavioral changes.

## Figures and Tables

**Table 1:** Unrest index for each treatment between the levels of the variables: period of the day, production cycle, and thermal comfort.

		Treatment		
		Blue	Green	Red
Period of day	Morning	40.0 ^Aa^	34.7 ^Ab^	32.0 ^Bc^
	Afternoon	38.3 ^Ba^	31.8 ^Bc^	33.4 ^Ab^
Comfort	Cold Stress	38.1 ^Ba^	31.2 ^Bc^	35.2 ^Ab^
	Comfort	40.7 ^Aa^	35.3 ^Ab^	31.5 ^Bc^
	Heat Stress	38.6 ^Ba^	33.2 ^Ab^	31.4 ^Bc^

Lowercase letters ^(^^a,b)^ indicate differences between the lighting treatments (columns) and uppercase letters ^(A,B)^ indicate differences between the lines of the same variable, by the Tukey test at 5% significance.

**Table 2:** Cluster index for each treatment between the levels of the variables: period of the day, production cycle, and thermal comfort.

		Treatment		
		Blue	Green	Red
Period of day	Morning	3.16 ^Ab^	3.15 ^Ab^	6.18 ^Aa^
	Afternoon	2.82 ^Bb^	2.54 ^Bb^	2.96 ^Ba^
Comfort	Cold Stress	3.07 ^b^	2.64 ^Bc^	4.66 ^Aa^
	Comfort	2.98 ^b^	2.92 ^Ac^	4.79 ^Aa^
	Heat Stress	2.91 ^b^	2.98 ^Ab^	4.26 ^Ba^

Lowercase letters ^(^^a,b)^ indicate differences between the lighting treatments (columns) and uppercase letters ^(A,B)^ indicate differences between the lines of the same variable, by the Tukey test at 5% significance.

## Data Availability

Data will be available upon request to the corresponding author.
